# Wild Mushrooms: A Hidden Treasure of Novel Bioactive Compounds

**DOI:** 10.1155/2023/6694961

**Published:** 2023-09-22

**Authors:** Gebreselema Gebreyohannes, Desta Berhe Sbhatu

**Affiliations:** Department of Biological and Chemical Engineering, Mekelle Institute of Technology, Mekelle University, Mekele, Ethiopia

## Abstract

Secondary metabolites are hidden gems in mushrooms. Understanding these secondary metabolites' biological and pharmacological effects can be aided by identifying them. The purpose of this work was to profile the mycochemical components of the extracts of *Auricularia auricula judae*, *Microporus xanthopus*, *Termitomyces umkowaani*, *Trametes elegans*, and *Trametes versicolor* to comprehend their biological and pharmacological capabilities. Mushroom samples were collected from Kenya's Arabuko–Sokoke and Kakamega National Reserved Forests and identified using morphological and molecular techniques. Chloroform, 70% ethanol, and hot water solvents were used to extract the mycochemical components. Gas chromatography mass spectrometry (GC-MS) was used to analyze the chloroform, 70% ethanol, and hot water extracts of all the species examined. A total of 51 compounds were isolated from all extracts and classified as carboxylic acids, esters, phenols, fatty acids, alcohol, epoxides, aldehydes, fatty aldehydes, isoprenoid lipids, and steroids. Tetracosamethyl-cyclododecasiloxane (18.90%), oleic acid (72.90%), phenol, 2, 6-bis (1, 1-dimethylethyl)-4-methyl-, and methylcarbamate (26.56%) were all found in high concentrations in *A. auricular judae*, *M. xanthopus*, *T. umkowaani*, *T. elegans*, and *T. versicolor*, respectively. Fatty acids make up the majority of the compounds isolated from the *T. elegans* chloroform extract and the *T. umkowaani* 70% ethanol extract, respectively. Particularly, these fatty acids play crucial roles in the anti-inflammatory, hypocholesterolemic, anticancer, and antibiofilm formation activities. These bioactive elements indicate that the extracts of five wild mushrooms may be reliable sources of secondary metabolites for therapeutic development. Therefore, additional research is required to comprehend the usefulness of these chemicals in many functional areas and to improve the present understanding of macrofungi.

## 1. Introduction

Macrofungi are sources of a wide range of physiologically active substances [1, 2]. Despite being a significant source of numerous bioactive chemicals that can be used to produce innovative medications, macrofungi have not yet been extensively utilized [3]. A growing area of research is the hunt for fungus-derived bioactive substances [4]. In light of treatment failures and the global multidrug resistance crisis, a constant hunt for new molecules with therapeutic value has become imperative [5]. For downstream applications and bioprospecting, mushrooms have a wide range of compounds [6]. Natural chemicals obtained from mushrooms can be thoroughly investigated and evaluated, which can be extremely beneficial for treating both infectious and noninfectious disorders [7, 8]. Medicinal mushrooms and fungi are believed to have 130 different therapeutic properties, including antiallergic [9], antiarthritic, antiasthmatic [10], anticancer [11], pesticide [12], antidepressive [13], antidermatophytic [14], antidiabetic [15], antifertility, antifungal [15], antihelminthic [16], cytotoxic [17], antihypercholesterolemic [18], antihyperlipidemic [19], antihypertensive [20], antihypocholesterolemic [21], anti-inflammatory [15], antimalarial [22], antimicrobial [15], antioxidant [11], antiparasitic [23], antispasmodic [24], antiviral [13], anticardiovascular illnesses [18], hepatoprotective [10], immunomodulator [22], immunostimulant [13], insecticidal [22], larvicidal [25], nematicide [26], nephroprotective, neuroprotective [13], osteoprotective [4], and vasodilator [27], among others.

*Auricularia auricular judge* (Bull.) belongs to phylum-basidiomycota, class: agaricomycetes, order: auriculariales, family: auriculariaceae, and genus: *Auricularia*. *A. auricula-judae*, also known as black fungus, wood ear, Jew's ear, or jelly ear, is a species of edible mushroom that is very nutrient-dense [28, 29]. It contains a variety of nutrients that are worthy for our health, including polysaccharides, melanin, polyphenols, flavonoids, amino acids, carbs, vitamins, and trace minerals. Furthermore, it has a variety of chemical combinations with antioxidant, anticoagulant, and anticancer properties [30].

*Microporus xanthopus* (Fr.) kuntze is a member of the genera *Microporus* and phylum-basidiomycota, class: agaricomycetes, order: polyporales, and family: polyporaceae. It is a polypore medicinal mushroom that cannot be eaten. It contains a variety of chemical components, including alkaloids, flavonoids, steroids, triterpenoids, and coumar, which have the potential to have positive pharmacological effects with applications in agriculture, medicine, and other fields [31]. According to reports, it has anthelmintic [32], antibacterial [33], anticancer [1], and antiangiogenic properties.

*Termitomyces umkowaani* is a member of the phylum-basidiomycota, class: agaricomycetes, order: agaricales, and family: lyophyllaceae. *Termitomyces* species are obligate mutualistic edible mushrooms that coexist with fungus-growing termites [34]. *Termitomyces*' geographic range matches that of termites, and it gives its hosts vitamins and digesting enzymes [35, 36]. *Termitomyces*' bioactive substances may be able to treat diseases including Alzheimer's, hyperlipidemia, cancer, and gastroduodenal disorders [34, 37].

*Trametes elegans*is a member of the phylum-basidiomycota, class: agaricomycetes, order: agaricales, and family: polyporaceae. *T*. *elegans* is an endophytic and saprotrophic fungus that brought white rot on wood [38]. Due to its extensive use in the culinary and pharmaceutical industries, *T. elegans* (also known as Turkey tail) has become incredibly well-known [39]. Its capacity to break down dead organic matter and utilize a variety of substrates has resulted in a diversity of its biological and metabolic processes [40]. It is also well-known for its therapeutic benefits, commercial applications (such as in the food sector), and roles in bioremediation and the biodegradation of cellulosic waste [41, 42].

Numerous biologically active polysaccharides found in *Trametes versicolor* (L) Lloyd (family: polyporaceae) are used to treat a variety of ailments, including rheumatoid arthritis, chronic hepatitis, infections of the respiratory tract, urinary tract, and digestive system, and tumors. It consists of 18 different amino acids, including aspartic acid, threonine, serine, glutamic acid, glycine, alanine, valine, and leucine, as well as a wide range of other substances, including proteins, fatty acids, polysaccharides, polysaccharopeptides, glucans, vitamins, and inorganic salts [43, 44]. All of these amino acids are necessary for growth and repair because they play various roles in cellular, tissue, and organ structure [45, 46].

Only a small or nonexistent amount of research has been carried out on the identification of bioactive chemicals that confer these therapeutic capabilities on the very few species of Kenyan wild mushrooms that have been claimed to have therapeutic potential. Determining the bioactive substances in wild mushroom extracts that are responsible for their therapeutic benefits is crucial. Therefore, the objective of this study was to investigate the bioactive substances found in the chloroform, ethanol, and hot water extracts of five wild mushrooms and to ascertain their biological and pharmacological therapeutic capabilities that may shed light on their usage in both traditional and contemporary medicine.

## 2. Materials and Methods

### 2.1. Wild Mushrooms Collection and Identification

In the national reserved forests of Kakamega and Arabuko–Sokoke, mushrooms were collected. They were randomly collected from tree bark or other substrates (wood, soil, or leaf litter). To keep them structurally sound and wet, they were wrapped in aluminum foil and put in an icebox. Following that, they were recognized using both morphological and molecular techniques. Specimens were identified using spore print color (white, black, brown, pink, purple, etc.) and macroscopic and microscopic methods (form and size of basidiospores, basidia, cystidia, and generative hyphae) [47]. In addition, *Species Fungorum* and associated literature were used to compare the morphological traits of the specimens [48]. The size and form of the gill, the color and shape of the cap, the color and shape of the stipe, and other morphological characteristics of the mushroom are some of the parameters utilized for identification. For the sake of identification, the gill margin, stipe location, stipe base, and pileal margin and surface were also applied. Other morphological details of the mushrooms, such as the cap's structure, the gills' margin and placement, the stipe's surface and form, and the pileus' margin and surface, were also noted. The ornamentation of the pileus and stipe surfaces, the presence or absence of an annulus on the stipe, and the presence or absence of a volva at the base of the stipe were additional characteristics that were utilized to describe and identify the mushrooms (Table 1). The samples were then preserved for additional analyses after being dried in an electric drying oven at 50°C for 168 h [49].

The dried fruiting body of mushrooms was used to extract gDNA using the cetyl trimethyl ammonium bromide (CTAB) technique [12]. Highly conserved portions of the ITS1 and ITS4 of the mushroom rDNA genes were amplified using the PCR amplification technique by using specialized markers [50]. PCR products that had been amplified were separated using gel electrophoresis and seen under UV illumination. Each PCR product's presence and quantity were calculated by contrasting it with the control (1 kb DNA ladder).

### 2.2. Extraction of Bioactive Compounds

Chloroform, 70% ethanol, and hot water solvents were used to extract bioactive chemicals, with a few changes from other investigations [51, 52]. A 100 g of powdered mushroom was combined with 1L of each of distilled hot water (heated at 60°C for 2 h.), 99.8% chloroform (Sigma Aldrich, USA), and 70% ethanol (ECP Ltd, New Zealand) separately in an Erlenmeyer flask at 25°C and shaken using an incubator shaker (SK-727, Amerex Instruments, Inc., USA) at 150 rpm for 72 h. The extracts were concentrated and dried using a rotary evaporator (EV311, Lab Tech Co., LTD, UK) at 50°C after being centrifuged at 3000 rpm for 15 min (Eppendorf centrifuge 5810 R, Germany). The extracts were freeze-dried (mrc freeze dryer, Model, FDL-10N-50-8M) and stored in a −80°C deep freezer. Finally, unprocessed extracts were placed in amber-colored bottles and placed in a refrigerator at +4°C for further analysis.

### 2.3. GC-MS Analysis of Extracts

The GC-MS analysis was conducted using a silica capillary column (30 × 0.25 mm ID × 1 *µ*m, composed of 100% dimethylpolysiloxane) and operated in an electron impact mode at 70 eV (Agilent Scientific, Palo Alto, CA). Helium (99.999%) was a carrier gas at a constant flow of 1 mL/min. Extracts were dissolved in dichloromethane and 1 *µ*L solution was injected into the column at 250°C and ion-source temperature 280°C. The oven temperature was programmed at 110°C for 2 min. The temperature was increased from 110°C to 200°C (10°C/min) then to 280°C (5°C/min) and finally ended at 280°C for 9 min. The total run time was 28 min. The compounds were identified from the MS data, by comparing the spectra of known compounds stored in the National Institute of Standards and Technology (NIST) library with the mass spectrometry (MS) of unknown compounds. The relative % amount of each compound was calculated by comparing its average peak area to the total areas. Measurement of peak areas and data processing were carried out by the Turbo-Mass-OCPTVS-Demo SPL software [53].

### 2.4. Statistical Analysis

All the tests, experiments, and measurements were carried out in triplicate. Microsoft Excel software package was used to analyze quantitative data [52].

## 3. Results and Discussion

### 3.1. GC-MS Analysis of Wild Mushroom Extracts

Five extracts of wild mushrooms were analyzed using GC-MS, and fifty-one (51) chemicals were found. Some of the compounds obtained are acyclic monoterpenoids, alcohol, aldehyde, alkene, alkyl benzene, aromatic organic heterocyclic, benzoic acid ester, cycloalkane methanol, cyclohexane, epoxides, ester, fatty acid, fatty acid ester, fatty alcohol, fatty aldehyde, isoprenoid lipid, organosiloxane, phenol, phthalate, pyrrolidines, siloxane, steroid, and *β*-carotene. Tables 2–6 list the many compounds and the pharmacological and biological actions of each one.

#### 3.1.1. GC-MS Analysis of *Auricularia auricula-judae*–AAJ

**Figure 1:**
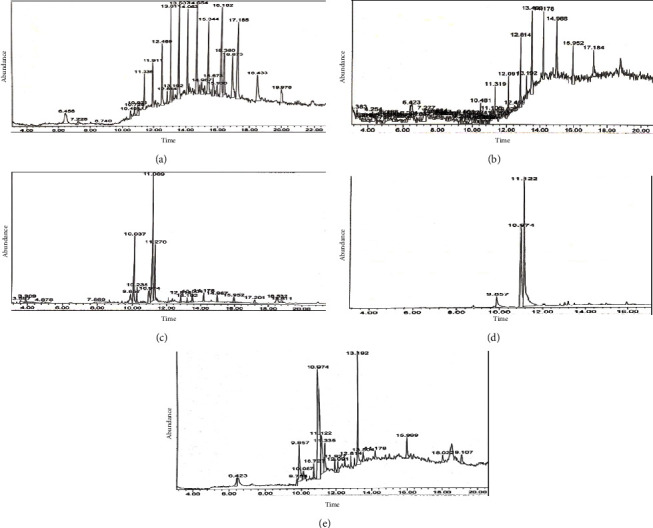
GC-MS chromatogram of five mushroom extracts. (a) Hot water extract of *A. auricula judae*. (b) Hot water extract of *M. xanthopus*. (c) 70% ethanol extract of *T. umkowaani*. (d) Chloroform extract of *T. elegans*. (e) Hot water extract of *T. versicolor*.

The hot water extract (HWE) of AAJ found fourteen (14) bioactive chemicals, as shown in Figure 1(a). These substances have shown a wide range of biological and pharmacological functions. Phenol, 2,6-bis (1,1-dimethyl ethyl)-4-methyl-, methylcarbamate (14.21%), 2-nonanol, 5-ethyl- (11.34%), octasiloxane, 1,1,3,3,5,5,7,7,9,9,11,11,13,13,15,15-hexadecamethyl- (10.65%), 2-methyl-6-methylene-octa-1,7-dien-3-ol (7.98%), 2-methyl-1-ethylpyrrolidine (7.23%), salicylic acid, and diethyl bis (trimethylsilyl) ester (7.12%) were identified as major compounds Table 2. These compounds were classified into 1-heptanol, 2,4-dimethyl- (R, R)- (+)- (alcohol), 1-hexene, 4, 5-dimethyl- (alkene), octasiloxane, 1,1,3,3,5,5,7,7,9,9,11,11,13,13,15,15-hexadecamethyl- (siloxane), Carbonic acid, methyl octyl ester and salicylic acid, diethyl bis (trimethylsilyl) ester (ester), di-n-octyl phthalate (phthalic acid), and di-n-decylsulfone (phthalate). The fruiting body of AAJ is reported to contain large amounts of fiber, carotenes, minerals (calcium, phosphorous, and iron), and vitamins in addition to proteins, carbs, and lipids [28]. In addition, polysaccharides, melanin, and polyphenols—vital categories of secondary metabolites that are synthesized in response to biotic (pathogens) and abiotic stresses—salinity, water, and climatic stress—are present in AAJ as bioactive constituents [28]. According to one study, siloxanes have been well-acknowledged to possess substantial antibacterial and antioxidant effects [54]. Thus, the compounds (di-n-decyl sulfone, cyclohexano, 2,4-dimethyl-, salicylic acid, diethyl bis (trimethylsilyl) ester, carbonic acid, methyl octyl ester, phenol, 2,6-bis (1,1-dimethylethyl)-4-methyl-, methylcarbamate, etc.,) found in the HWE of AAJ could prevent diseases such as aging, cancer, cardiovascular disease, inflammation, and other disorders that are dangerous to humans' health occurred due to the overabundance of free radicals in our body [108]. In addition, phenolic compounds can influence biological processes such as cell cycle control, apoptosis induction, and antiproliferation, which are primarily mediated through interactions between receptors and ligands [92].

Many biological and pharmacological activities, including antidepressant, antimicrobial, antioxidant, antimalarial, anti-inflammatory, insecticidal, hepatoprotective, antihelminthic, larvicidal, antihypertensive, anticancer, antidiabetic, cholesterol-lowering, antiurolithiasis, and antifertility, have been demonstrated by the HWE of AAJ, as shown in Table 2. The anticoagulant, antidiabetic, antioxidant, anticancer, hypolipidemic, antiobesity, anti-inflammatory, antiradiation, immunomodulatory, and antibacterial properties of AAJ extracts have also been established by earlier investigations [28, 109, 110]. According to a study, the phenolic substances epicatechin, catechin, chlorogenic acid, quercetin, and rutin are among the phenolic compounds found in the HWE of AAJ. Significant scavenging ability was shown by these phenolic compounds against hydroxyl radicals, superoxide anions, and DPPH-free radicals [111]. Crude AAJ extracts have greater antioxidant activity, control blood pressure, and reduce blood lipid and cholesterol levels [28].

Octasiloxane, 1,1,3,3,5,5,7,7,9,9,11,11,13,13,15,15-hexadecamethyl, salicylic acid, diethyl bis (trimethylsilyl) ester, di-n-octyl phthalate, di-n-decyl sulfone, carbonic acid, and methyl octyl ester have all demonstrated antibacterial action in this study. The antibacterial activity of crude polysaccharides derived from AAJ has been demonstrated in the past against *E. coli*, *S. aureus*, *B. cereus*, *S. typhi*, *P. mirabilis*, *K. pneumoniae*, *P. aeruginosa*, *C. albicans*, and *C. parapsilosis* [28, 109]. Many secondary metabolites, including *β*-glucans, chitin, and derivatives of the sterol ergosterol, have been detected in numerous *in vitro* and *in vivo* studies. These metabolites demonstrate potential anti-inflammatory activity by reducing the production of proinflammatory cytokines, promoting the production of anti-inflammatory cytokines, and preventing both immune response and the development of cancer cells in the body [28, 30, 110, 112]. They also protect the body by lowering blood cholesterol, boosting our immune system, preventing inflammatory disorders, and delaying the development of cancer [28, 112, 113].

By regulating pancreatic insulin secretion, mushroom polysaccharides have demonstrated antidiabetic properties that help to maintain blood glucose homeostasis [114]. A prior study claimed that polysaccharides derived from AAJ extracts significantly reduced the risk of diabetes in streptozotocin-induced diabetic rats. After giving AAJ polysaccharides to streptozotocin and high-fat diet-induced diabetic rats, low-density lipoprotein, and total cholesterol levels in the blood were markedly decreased [115]. In addition, streptozotocin-induced diabetic mice improved the insulin resistance islet damage in diabetes-induced rats treated with AAJ polysaccharides, which changed glucose metabolism, elevated insulin levels, and decreased blood glucose levels [116, 117]. These results supported the idea that AAJ-derived polysaccharides could be employed as possible diabetic treatment agents by modifying blood glucose levels [28].

High concentrations of insoluble fibers can be found in the HWE of AAJ [118]. Through the modification of gut microbiota, these fibers may have positive effects on health [119, 120]. Insoluble fibers have a crucial role in regulating the environment of the gut microbiota and influencing their metabolic activities [121, 122]. They also serve as prebiotics. By fighting for food and preventing adhesion to the gut wall, the beneficial gut microbiota is essential in defending our body against a variety of disease-causing pathogenic bacteria [123]. These gut bacteria also aid in the formation of short-chain fatty acids, such as acetate, propionate, and butyrate, which are crucial for our epithelial cells during their digestion and fermentation processes [124, 125]. By upholding a healthy gut environment and acting as the only carbon source for intestinal bacteria during fermentation, *β*-glucans derived from HWE of AAJ have many health-promoting effects. They also raised the quantity of good bacteria, such as *Bifidobacteria* and *Lactobacillus*, which aid in the synthesis of short-chain fatty acids in our intestines [126]. During the oral treatment of mice, they also saw an increase in serum IgA and IgG levels [127]. Furthermore, they stop the development of harmful bacteria in our gut, which may eventually shield our bodies against a variety of disorders linked to the gut [128, 129].

Biological defenses against cardiovascular disease exist in edible mushrooms. There have been claims that certain *Auricularia* species contain substances that decrease cholesterol [130]. AAJ extracts have reportedly been shown to lower low-density lipoprotein cholesterol levels, which are the cause of cardiovascular disease [121]. AAJ extract effectively decreased serum and liver total cholesterol (TC), total triglyceride (TG), and serum lactate dehydrogenase C (LDH-c) levels in mice using hyperlipidemic mice as a model [131].

Natural immunomodulators can be found in large quantities in medicinal mushrooms. They contain a variety of immune-regulatory substances, including immunomodulatory proteins, lectins, polysaccharides, and terpenes. Immunomodulators can act as immunological adjuvants, immune stimulants, or immune suppressants [132]. For instance, an active substance from AAJ called AF1 with a 1, 3-d-glucan main chain and two 1, 6-d-glucosyl residues has been shown to cause apoptosis in cancer cells [133].

#### 3.1.2. GC-MS Analysis of Hot Water Extract of Microporusxanthopus–MX

Twelve chemicals were found in the hot water extract (HWE) of *M. xanthopus* (MX), as shown in Figure 1(b).  Table 3 lists the twelve compounds and their relative abundances. These include trans-1, 1′-bibenzoindanylidene (14.18%), 2, 2′-divinylbenzophenone (13.76%), and didodecyl phthalate (11.39%). Alcohol, epoxides, aldehydes, fatty aldehydes, isoprenoid lipids, n-alkanes, and steroids were the several classifications given to the substances. These substances have been demonstrated to have antioxidant, antimicrobial, nematicidal, antimalarial, antidiuretic, antiasthmatic, vasodilator, antifouling, antidermatophytic, antihypertensive, uric acid excretion stimulant and diuretic, lowering depressed symptoms, and anti-inflammatory properties. In addition, the steroid 1-monolinoleoylglycerol trimethylsilyl ether exhibits antidiuretic, antidiabetic, anti-inflammatory, antimicrobial antioxidant, antiarthritic, and antiasthma properties Table 3.

In line with the current findings, HWE of MX, numerous mushroom extracts including *Agaricus bisporus*, *Cyclocybe aegerita*, *Cyclocybe cylindracea*, and *Tremella fuciformis* have been investigated for the treatment or prophylaxis of type-2 diabetes, which develops when insulin production is unbalanced as a result of the dysfunction of insulin-secreting beta cells in the pancreas [134, 135]. Mushrooms assist patients in avoiding excessive blood sugar levels since they are the food with the least quantity of digestible carbohydrates [136]. Diabetes can be treated with bioactive compounds that are extracted from medicinal mushrooms [137, 138]. Extracts from *Inocutis levis* and *Antrodia cinnamomea* have been suggested as treatments for diabetes because they improve insulin resistance, insulin sensitivity, and tissue uptake of glucose, which helps to regulate blood sugar levels [135, 139].

Most of the substances discovered in the HWE of MX in the current results demonstrated antibacterial action. Another investigation confirmed that HWE of MX-derived oligosaccharides, polysaccharides, and polyphenols had antibacterial effects on *S. aureus* strains that were resistant to methicillin and *E. coli* strains that produced Shiga toxin [33]. Similar to this, MX's CE has shown increased antibacterial activity against *S. aureus* (ATCC 25923), MRSA (ATCC 33591), and *K. pneumoniae* (ATCC 13883) [52].

The majority of mushrooms are known to produce several bioactive compounds that are employed as potential treatments for cardiovascular disorders [140, 141]. Although the mechanism of action/treatment of these bioactive compounds is still unknown, it may be related to changes in phospholipid metabolism, bile acid secretion, and LDL receptor expression [142]. The presence of chemicals in mushrooms that can alter cholesterol metabolism, absorption, and gene expression has also been noted in other investigations [141, 143]. For example, *Grifola frondosa*, *Hypsizigus marmoreus*, and *Pleurotus ostreatus* extracts have been shown to alter the gene expression patterns in mouse livers [135, 144].

#### 3.1.3. GC-MS Analysis of 70% Ethanol Extract of *Termitomyces umkowaani* (TU)

The 70% ethanol extract (EE) of *T. umkowaani* (TU) (Figure 1(c)) was used to identify fourteen different components. These substances were categorized into acids, alcohols, esters, ethers, ketones, aldehydes, and other categories. Tetracosamethyl-cyclododecasiloxane (18.90%), 12-methyl-E, E-2, 13-octadecadien-1-ol (15.90%), 9, 12-octadecadienoic acid, and ethyl ester (13.43%) were the most prominently seen compounds Table 4.

Many fatty acids (FAs) such as linolenic acid, butanedioic acid diethyl ester, octadecanoic acid, ethyl ester, h-hexadecanoic acid, hexadecanoic acid, ethyl ester, i-propyl hexadecanoate, 9, 12-octadecadienoic acid (Z, Z)-, 9, 12-octadecadienoic acid, ethyl ester, and 7-hexadecenal, (Z)- were noticed in EEofTU. These FAs demonstrated antimicrobial, pesticide, antioxidant, antispasmodic, antitumor, antihypocholesterolemic, antiarthritic, anti-inflammatory, nematicide, immunostimulant, antiacne, insecticide, antieczemic, hepatoprotective, antihistaminic, and anticoronary properties [145–147]. In addition to FAs, the EE of TU revealed additional bioactive substances, such as isopropyl linoleate (-carotene), 1-monolinoleoylglycerol trimethylsilyl ether (steroid), and 12-methyl-E, E-2, 13-octadecadien-1-ol (alcohol). These substances also have antibacterial, antioxidant, antiasthma, antidiuretic, anti-inflammatory, and antidiabetic activities. By preventing the proliferation of bacterial cells and the development of biofilms, linoleic and oleic acids displayed an antibacterial activity against *S*. *aureus* [148].

The EE of TU contains hexadecanoic acid, and ethyl ester (palmitic acid ester), which has antioxidant, hypocholesterolemic, nematicide, pesticide, antiandrogenic, antibacterial, anti-inflammatory, antitumor, immunostimulant, hemolytic 5-reductase inhibitor, and lipoxygenase inhibitor effects. Dietary fats contain palmitic acid (PLA), which ensures an intake of 20 g on average per day. Its important nutritional function [149] justifies the relatively high need for these fatty acids in the human body (20–30% of total fatty acids). It was discovered through transcriptomic research that palmitic acid affected many signaling pathways, including lipid metabolism in neurons. On the other hand, excessive ingestion of palmitic acid has been linked to neurodegenerative conditions, such as Parkinson's disease [150, 151]. To prevent harmful effects such as oxidative stress, PLA has a critical role in low levels of stress, which can activate the stress response pathway.

The EE of TU contained the 9, 12-octadecadienoic acid (Z, Z)-, that is also known as conjugated linoleic acid. Omega-3 and omega-6 fatty acids are present in linolenic acid (LA). LA can minimize risk factors for arthritis and heart disease as well as assist in reducing bodily inflammation. Prostaglandin E1, a product of omega-3 fatty acids, increases immunity and lowers blood cholesterol [152, 153]. The heart's health is improved by omega-3 fatty acids, which also lower the risk of stroke, heart attack, and high blood pressure [153, 154]. In general, mushrooms have more unsaturated fatty acids than saturated ones [155]. These polyunsaturated fatty acids preserve the liver's ability to produce bile acids, prevent hormonal imbalance, and affect prostaglandin synthesis [156].

LA currently exhibits antibacterial action. In support of the present findings, *Termitomyces* species extract in methanol and ethanol demonstrated strong antibiotic action against pathogenic microorganisms such as *E. coli, B. cereus*, *S. aureus*, *P. aeruginosa*, *S. typhimurium*, and *C. albicans* [157]. The dichloromethane extract of *Termitomyces striatus* also demonstrated antibacterial action against fungi (*C. albicans* and *S. cerevisiae*) and bacteria (*P. aeruginosa*, *E. coli*, *B. subtilis*, and *S. aureus*) [158]. Numerous species of *Termitomyces* have notable antibacterial action against various harmful pathogens. For instance, the water extract of *T. clypeatus* demonstrated antibacterial and antifungal activities against *C. albicans*, *E. coli*, *S. typhi*, and *S. aureus*. The water extract of *T. heimii*also demonstrated antibacterial and antifungal activities against *E. coli*, *K. pneumoniae*, *Pseudomonas* sp., *S. aureus*, *S. pyogenes*, and *Ralstonia* sp.

Fatty acids from the EE of TU, such as octadecanoic acid, ethyl ester, h-hexadecanoic acid, 9, 12-octadecadienoic acid (Z, Z), 9, 12-octadecadienoic acid, and ethyl ester, have demonstrated hypocholesterolemic action. Edible mushrooms include large levels of dietary fiber and other nutrients such as eritadenine, guanylic acid, and ergosterol that can prevent diseases associated with nutrition, including atherosclerosis, by reducing hypocholesterolemic levels [159, 160]. Total and LDL cholesterol levels in the blood were found to decrease with dietary TU intake [161]. Rats that were fed diets containing a combination of mushrooms saw a decrease in their triglyceride and total cholesterol levels [156]. By changing lipid metabolism and preventing both the buildup of liver lipids and the rise of serum lipids, polysaccharides and fibers extracted from aqueous extract of edible mushrooms also reduced the serum triglyceride concentration inhypertensive and hyperlipidaemic rats [162].

#### 3.1.4. GC-MS Analysis of Chloroform Extract of *Trametes elegans* (TRE)

Three substances were found in the *T. elegans* chloroform extract (CE), as shown in Figure 1(d). N-hexadecanoic acid (16.89%), oleic acid (72.90%), and octadecanoic acid (10.21%) are among the chemicals that have been discovered. These substances fall under the category of essential fatty acids, which are required for their anti-inflammatory, antioxidant, and hypocholesterolemic properties. In rats, a lack of the normal necessary fatty acid linoleic acid results in hair loss [21], minor skin scaling, and slow wound healing [22].

According to Table 5, the majority of the discovered compounds show antimicrobial, antioxidant, anticancer, antiandrogenic, hypocholesterolemic, nematicide, pesticide, and antibiofilm formation capabilities. Numerous substances found in the extract, including tocopherols, flavonoids, polyphenols, tannins, and lignins, may be associated with these wide-ranging activities [163]. The oxidizing cascade of free radical reactions in molecules is blocked by the TRE extract's antioxidant activity, which also lessens oxidative damage brought on by oxidative stress [164]. Antioxidants shield our bodies from serious health problems such as diabetes, cancer, aging, atherosclerosis, and others [165].

From the CE of TRE, three isolated essential fatty acids have shown antibiofilm-forming activity. By preventing pathogenic microorganisms from forming biofilms, fungal metabolites have antiquorum-sensing properties that have the potential to reduce the development of drug resistance. Numerous secondary metabolites with biofilm-inhibitory properties are found in many edible mushrooms, according to earlier investigations. For instance, active antibiofilm inhibitory activity against *Pseudomonas*, *S. aureus*, and *C. albicans* has been demonstrated by coprinuslactone, roussoellenic acid, and microporenic acid A obtained from *Coprinus comatus*, *Roussoella* sp, and Kenyan basidiomycete, respectively [166, 167]. By facilitating antibiotics' capacity to enter biofilms, biofilm inhibitors improve the effectiveness of the antibiotics [168].

The CE of TRE possesses anticancer properties. The extracts combat cancer cells in a variety of ways, such as immune system regulation and cell death [169]. Preclinical and clinical testing for several promising anticancer drugs based on fungi is now underway [170]. Irofulven, for instance, is a semisynthetic medication made from illudin S, a toxin discovered in the *Omphalotus illudens*. Irofulven has been tested in phases I and II clinical studies, showing promise in its ability to treat malignancies of the breast, blood, colon, sarcoma, prostate, lungs, ovary, and pancreas, as well as the brain and central nervous system [171, 172]. Another anticancer substance discovered in fungi *Akanthomyces muscarius* and *Nigrospora sphaerica* is called aphidicolin. Aphidicolin has not yet been marketed as an anticancer medication, even though it specifically targets the binding site on the DNA polymerase and enzymes [135].

One of the fatty acids, n-hexadecanoic acid, found in the CE of TRE showed nematicidal action as shown in Table 5. Even though chemical nematicides (like methyl bromide) are effective and have been commercialized, they can have detrimental effects on the environment by destroying all soil life and thinning the ozone layer. Finding ecologically sound substitutes has recently been the focus of intense research in both academia and industry [135]. Edible mushrooms have been found to contain several nematotoxic substances, including fatty acids, alkaloids, peptide compounds, terpenes, condensed tannins, phenolic compounds, and proteases [173]. One of the nematicidal substances that have been identified from *Arthrobotrys* species and other fungi is linoleic acid [174]. *Pleurotus pulmonarius* and *Hericium coralloides*, on the other hand, are two basidiomycetes that have demonstrated potent nematicidal actions against *Caenorhabditis elegans* [175]. From a *Sanghuangporus* species obtained in Kenya, metabolites (3, 14′-bihispidinyl and hispidin and phelligridin L) with mild nematicidal activity against *Caenorhabditis elegans* have been identified [176]. Recent studies have shown that chaetoglobosin A and its derivative 19-O-acetylchaetoglobosin A, which were isolated from *Ijuhya vitellina*, have nematicidal activity against the eggs of *Heterodera filipjevi* [177].

#### 3.1.5. GC-MS Analysis of Hot Water Extract of *Trametes versicolor* (TRV)

*T. versicolor* (TRV) hot water extract (HWE) was used to identify eight different chemicals (Figure 1(e)). The most prevalent substances were phenol (26.56%), 2, 6-bis (1, 1-dimethyl ethyl)-4-methyl, methylcarbamate (22.40%), 1, 2-benzene dicarboxylic acid, and diisooctyl ester (19.10%), as shown in Table 6.

In TRV, a polyunsaturated fatty acid called 9, 12-Octadecadienoic (Z, Z)- has demonstrated antitumor action [107]. The TRV extract contains substances that fight cancer and boost the immune system, such as polysaccharides, *β*-glucans, lignins, and ergosta-7, 22-dien-3 beta-ol [178]. Cytotoxic action against cancer cells was shown by polysaccharides extracted from the TRV extract [45]. In addition to significantly improving the quality of life of cancer patients receiving chemotherapy or radiation therapy, polysaccharides containing peptides also help patients with hepatitis, hyperlipidemia, and other chronic diseases live longer and have better quality of life [178, 179]. In mice bearing xenografts, an aqueous extract of TRV inhibited the migration and invasion of 4T1 breast cancer cells and downregulated the activity of the xenograft-inducing molecules tumor necrosis factor, interferon, interleukin-2, interleukin-6, and interleukin-12 [180]. The polysaccharides linked to the TRV protein displayed tumor necrosis factor-dependent antiproliferative activity towards MCF-7 cells and enhanced the proliferative response of blood lymphocytes, which was connected to the upregulation of interleukin-6 and interleukin-1 mRNA [181].

## 4. Conclusion

Bioactive compounds identified from the five wild mushroom extracts possess anti-inflammatory, antioxidant, nematicide, antimicrobial, anticancer, hypocholesterolemic, antihypertensive, pesticide, and antibiofilm formation properties. The wild mushroom extracts are rich in essential fatty acids and other many bioactive compounds that could have high industrial potential and biological activities. Phenol, 2, 6-bis (1, 1-dimethylethyl)-4-methyl-, methylcarbamate (*A. auricular-judae*), 1-monolinoleoylglycerol trimethylsilyl ether (*Microporus xanthopus*), tetracosamethyl-cyclododecasiloxane (*T. umkowaani*), oleic acid (*T. elegans*), and phenol, 2, 6-bis (1, 1-dimethylethyl)-4- methyl, methylcarbamate (*T. versicolor*) are the most abundant compounds. These compounds can be deployed to discover novel drugs against various noninfectious diseases such as cancer, hypertension, and diabetes. The identified compounds shall be subjected to further studies to utilize their usefulness in the prevention and treatment of infectious and noninfectious human diseases. To understand the mechanisms of action of the active ingredients, rigorous chemical analyses as well as *in vivo* pharmacokinetics and pharmacodynamics of individual compounds are needed. Future investigation is needed to clarify the long-term effects of taking medicinal mushroom products with other drugs.

## Figures and Tables

**Table 1:** Morphological characters and keys used for the identification of the wild mushrooms.

Mushrooms species	Cap structure	Location of stipe	Pileus margin	Pileal shape	Pileal surface	Annulus (veil/ring)	Volva (cup)	Stipe surface	Stipe base/shape	Gill margin	Gill attachment
*Auricularia auricula judae*	N/A	N/A	Incurved	Ear shaped	N/A	—	—	N/A	No stipe	N/A	N/A
*Microporus xanthopus*	Half funnel	N/A	Smooth	Hemispherical	Smooth	—	—	N/A	Substipitate	N/A	Sinuate
*Termitomyces umkowaani*	Umbonate	Central	Smooth	Knobbed	Smooth	—	—	Smooth	Swollen	N/A	Adnexed
*Trametes elegans*	Funnel-like	N/A	Smooth	N/A	Rough	—	—	N/A	Nonstipe	N/A	Adnate
*Trametes versicolor*	N/A	N/A	N/A	N/A	N/A	—	—	N/A	Substipitate	N/A	Free

*Note*. N/A-not applicable, — denotes the absence of annulus and volva.

**Table 2:** GC-MS analysis of *A. auricula judae* hot water extract.

Peaks	RT (min)	PA (%)	IUPAC name and MF of compounds	Nature of compounds	Pharmacological and biological activities	Ref.
1	19.98	10.65	Octasiloxane, 1,1,3,3,5,5,7,7,9,9,11,11,13,13,15,15-hexadecamethyl- (**C**_**16**_**H**_**50**_**O**_**7**_**Si**_**8**_)	Siloxane	Antidepressant and antimicrobial	[54, 55]
2	18.43	7.12	Salicylic acid, diethyl bis (trimethylsilyl) ester (**C**_**10**_**H**_**28**_**O**_**4**_**Si**_**3**_)	Ester	Antioxidant, antimicrobial, antimalarial, and anti-inflammatory	[56, 57]
3	17.19	4.43	Di-n-octyl phthalate (**C**_**24**_**H**_**38**_**O**_**4**_)	Phthalic acid	Antimicrobial and insecticidal	[58, 59]
4	16.87	6.12	Di-n-decylsulfone (**C**_**20**_**H**_**42**_**O**_**2**_**S**)	Phthalate	Antimicrobial, anticancer, antihelminthic, antagonistic, and larvicidal	[60, 61]
5	16.38	7.98	2-Methyl-6-methylene-octa-1,7-dien-3-ol (**C**_**10**_**H**_**16**_**O**)	Acyclic monoterpenoids	No activity reported	
6	16.18	5.65	1-Heptanol, 2,4-dimethyl- (R, R)- (+)- (**C**_**9**_**H**_**20**_**O**)	Alcohol	Antifungal, antioxidant, and anticholinesterase	[62–64]
7	15.34	4.31	Cyclohexanol, 2,4-dimethyl- (**C**_**8**_**H**_**16**_**O**)	Cyclohexane	Anticancer	[65]
8	14.65	3.43	Carbonic acid, methyl octyl ester (**C**_**10**_**H**_**20**_**O**_**3**_)	Ester	Hepatoprotective, antihypertensive, antioxidant, antimicrobial, antidiabetic, cholesterol-lowering, antiurolithiasis, and antifertility	[66]
9	14.06	5.25	1-Allylcyclopropyl methanol (**C**_**7**_**H**_**12**_**O**)	Cycloalkane methanol	No activity reported	
10	13.64	7.23	2-Methyl-1-ethylpyrrolidine (**C**_**7**_**H**_**15**_**N**)	Pyrrolidines	Antitumor	[67]
11	13.01	6.33	Oxirane, 2,2′-(1,4-dibutanediyl) bis- (**C**_**8**_**H**_**14**_**O**_**2**_)	Epoxides	Antibacterial	[68]
12	12.47	11.34	2-Nonanol, 5-ethyl- (**C**_**11**_**H**_**24**_**O**)	Fatty alcohol	Anticancer	[69]
13	11.91	5.86	1-Hexene, 4, 5-dimethyl- (**C**_**8**_**H**_**16**_)	Alkene	Antimicrobial	[70]
14	11.34	14.21	Phenol, 2,6-bis (1,1-dimethyl ethyl)-4-methyl-, methylcarbamate (**C**_**17**_**H**_**27**_**NO**_**2**_)	Alkylbenzene	Antioxidant, antibacterial, anti-inflammatory, and temporarily treat pharyngitis	[71, 72]

MF: molecular formula; RT: retention time; PA: peak area.

**Table 3:** GC-MS analysis of *M. xanthopus* hot water extract.

Peaks	RT (min)	PA (%)	IUPAC name and MF of compounds	Nature of compounds	Pharmacological and biological activities	Ref.
1	6.42	8.11	1-Heptanol, 2,4-dimethyl-, (2S, 4R) -(-)- (**C**_**9**_**H**_**20**_**O**)	Alcohol	Antifungal	[62, 63]
2	7.28	4.34	Oxirane, 2,2′-(1,4-butanediyl) bias- (**C**_**8**_**H**_**14**_**O**_**2**_)	Epoxides	No activity reported	
3	10.48	3.67	3-Methyl-2-(2-oxopropyl) furan (**C**_**8**_**H**_**10**_**O**_**2**_)	Aldehyde	Antioxidant and antimicrobial	[73, 74]
4	11.32	5.50	7-Hexadecenal, (Z)- (**C**_**16**_**H**_**30**_**O**)	Fatty aldehyde	Antiviral and antibacterial	[75, 76]
5	12.09	7.87	1,2,3,3a-Tetrahydro-7-methyl-10-4-methylphenyl) benzo [c] cyclopenta [f] −1,2-diazepine (**C**_**20**_**H**_**20**_**N**_**2**_)	Aromatic organic heterocyclic	No activity reported	
6	12.81	4.41	Tetradecane, 2,6,10-trimethyl- (**C**_**17**_**H**_**36**_)	Isoprenoid lipid	Antifungal, antibacterial, and nematicidal	[77]
7	13.19	4.19	Heptacosane (**C**_**27**_**H**_**56**_)	N-alkanes	Antibacterial, antifungal, antioxidant, antimalarial, and antidermatophytic	[78, 79]
8	13.47	11.39	Didodecyl phthalate (**C**_**32**_**H**_**54**_**O**_**4**_)	Phthalate	Vasodilator, antihypertensive, uric acid excretion stimulant and diuretic, antimicrobial, and antifouling	[80, 81]
9	14.18	1.17	Acetamide, N-[3-(10,11-dihydro-5H-dibenzo [a,d] cyclohepten-5-ylidene)propyl] −2,2,2-triflouro-N-methyl (**C**_**21**_**H**_**20**_**F**_**3**_**NO**)	Unknown	Reducing depressive symptoms	[82]
10	14.97	13.76	2,2′-Divinylbenzophenone (**C**_**17**_**H**_**14**_**O**)	Unknown	Antimicrobial, anti-inflammatory, and antioxidant	[83]
11	15.95	14.18	Trans-1, 1′-bibenzoindanylidene (**C**_**18**_**H**_**16**_)	Unknown	No activity reported	
12	17.18	16.32	1-Monolinoleoylglycerol trimethylsilyl ether (**C**_**27**_**H**_**54**_**O**_**4**_**Si**_**2**_)	Steroid	Antidiuretic, anti-inflammatory, antidiabetic, antimicrobial, antioxidant, antiarthritic, and antiasthma	[84, 85]

MF: molecular formula; RT: retention time; PA: peak area.

**Table 4:** GC-MS analysis of *T. umkowaani* 70% ethanol extract.

Peaks	RT (min)	PA (%)	IUPAC name and MF of compounds	Nature of compounds	Pharmacological and biological activities	Ref.
1	4.88	5.68	Butanedioic acid diethyl ester (**C**_**8**_**H**_**14**_**O**_**4**_)	Fatty acid	Antimicrobial, antispasmodic, and anti-inflammatory	[86]
2	7.87	4.11	Octadecanoic acid, ethyl ester (**C**_**20**_**H**_**40**_**O**_**2**_)	Fatty acid esters	Hypocholesterolemic5-alpha-reductase inhibitor, lubricant, and antimicrobial	[87, 88]
3	9.86	2.45	h-Hexadecanoic acid (**C**_**16**_**H**_**32**_**O**_**2**_)	Fatty acid (aka palmitic acid)	Antioxidant, hypocholesterolemic, nematicide, pesticide, antiandrogenic, antibacterial, anti-inflammatory, antitumor, immunostimulant, hemolytic 5-*α* reductase inhibitor, and lipooxygenase inhibitor	[5, 89]
4	10.04	7.90	Hexadecanoic acid, ethyl ester (**C**_**18**_**H**_**36**_**O**_**2**_)	Fatty acid ester (akapalmitic acid ester)	Antioxidant, hypocholesterolemic, nematicide, pesticide, antiandrogenic, and hemolytic 5-*α* reductase inhibitor	[5]
5	10.24	8.78	i-Propyl hexadecanoate (**C**_**19**_**H**_**38**_**O**_**2**_)	Fatty acid	No activity reported	
6	10.97	9.98	9,12-Octadecadienoic acid (Z, Z)-(**C**_**18**_**H**_**32**_**O**_**2**_)	Fatty acid (aka conjugated linoleic acid)	Anti-inflammatory, antioxidant, hypocholesterolemic, antimicrobial, antitumor, insecticide, antiarthritic, antieczemic hepatoprotective, antiandrogenic, nematicide, antihistaminic, antiacne, hemolytic 5-*α* reductase inhibitor, and anticoronary	[5, 66, 89–91]
7	11.09	13.43	9,12-Octadecadienoic acid, ethyl ester (**C**_**20**_**H**_**36**_**O**_**2**_)	Fatty acid ester (aka omega-6)	Hypocholesterolemic, nematicide, antiacne, antiarthritic, hepatoprotective, antimicrobial, antiandrogenic, hemolytic 5-*α* reductase inhibitor, antihistaminic, anticoronary, and insecticide, antieczemic	[5, 56, 66]
8	11.27	0.89	Isopropyl linoleate (**C**_**21**_**H**_**38**_**O**_**2**_)	*β*-carotene	Antimicrobial and antioxidant	[31, 92–94]
9	13.19	1.50	1-Monolinoleoylglycerol trimethylsilyl ether (**C**_**27**_**H**_**54**_**O**_**4**_**Si**_**2**_)	Steroid	Antimicrobial, antiasthma, antidiuretic, antioxidant, anti-inflammatory, and antidiabetic	[84]
10	14.18	15.90	12-Methyl-E, E-2, 13-octadecadien-1-ol (**C**_**19**_**H**_**36**_**O**)	Alcohol	Antimicrobial	[95]
11	14.97	1.12	7-Hexadecenal, (Z)- (**C**_**16**_**H**_**30**_**O**)	Fatty aldehyde	Antiviral and, antibacterial	[75, 76]
12	15.95	3.60	1, 2-Benzenedicarboxylic acid, diisooctyl ester (**C**_**24**_**H**_**38**_**O**_**4**_)	Ester	Antimicrobial and antifouling	[96]
13	17.20	18.90	Tetracosamethyl-cyclododecasiloxane (**C**_**24**_**H**_**72**_**O**_**12**_**Si**_**12**_)	Siloxane	No activity reported	
14	18.53	5.76	Heptasiloxanehexadecamethyl (**C**_**16**_**H**_**48**_**O**_**6**_**Si**_**7**_)	Organosiloxane	No activity reported	

MF: molecular formula; RT: retention time; PA: peak area.

**Table 5:** GC-MS analysis of *T. elegans* chloroform extract.

Peaks	RT (min)	PA (%)	IUPAC name and MF of compounds	Nature of compounds	Pharmacological and biological activities	Ref.
1	9.86	16.89	n-Hexadecanoic acid (**C**_**16**_**H**_**32**_**O**_**2**_)	Fatty acid	Antioxidant, antiandrogenic, hypocholesterolemic, nematicide, pesticide, and antibiofilm formation	[89, 97]
2	10.97	72.90	Oleic acid (**C**_**18**_**H**_**34**_**O**_**2**_)	Fatty acid	Antioxidant, apoptotic activity in tumor cells, anticancer, and antibiofilm formation	[97, 98]
3	11.12	10.21	Octadecanoic acid (**C**_**18**_**H**_**36**_**O**_**2**_)	Fatty acid	Antimicrobial and antibiofilm formation	[97, 99]

MF: molecular formula; RT: retention time; PA: peak area.

**Table 6:** GC-MS analysis of *T. versicolor* hot water extract.

Peaks	RT (min)	PA (%)	IUPAC name and MF of compounds	Nature of compounds	Pharmacological and biological activities	Ref.
1	6.42	26.56	Phenol, 2,6-bis (1,1-dimethyl ethyl)-4- methyl, methylcarbamate (**C**_**17**_**H**_**27**_**NO**_**2**_)	Phenol	Antioxidant, antibacterial, anti-inflammatory, oral anesthetic/analgesic, and temporarily treat pharyngitis	[71, 72]
2	9.86	2.20	n-Hexadecanoic acid (**C**_**16**_**H**_**32**_**O**_**2**_)	Palmitic acid	Antioxidant, nematicide, pesticide, hypocholesterolemic, and antiandrogenic	[100]
3	10.73	3.40	Nonadecane (**C**_**19**_**H**_**40**_)	Hydrocarbon	No activity reported	
4	11.12	8.41	9,12-Octadecadienoic (Z, Z)- (**C**_**18**_**H**_**32**_**O**_**2**_)	Polyunsaturated fatty acid	Anti-inflammatory, hypocholesterolemic, antitumor, hepatoprotective, nematicide, insecticide, antibiofilm formation, antihistaminic, antieczemic, antiacne, hemolytic 5-*α* reductase inhibitor, antiandrogenic, antiarthritic, and anticoronary, antimicrobial	[84, 97, 101–103]
5	11.34	5.73	7-Hexadecenal, (Z)- (**C**_**16**_**H**_**30**_**O**)	Fatty aldehyde	Antiviral and antibacterial	[75, 76]
6	13.19	12.20	9,12,15-Octadecatrienoic acid, 2-[(trimethylsilyl) oxy]-1-[[(trimethylsilyl) oxy] methyl] ethyl ester (Z, Z, Z)- (**C**_**27**_**H**_**52**_**O**_**4**_**Si**_**2**_)	Polyunsaturated fatty acid	Antimicrobial, antioxidant	[104, 105]
7	15.97	22.40	1-Momolinoleoylglycerol trimethylsilyl ether (**C**_**27**_**H**_**54**_**O**_**4**_**Si**_**2**_)		Antimicrobial, antiasthma, antidiuretic, antioxidant, anti-inflammatory, and antidiabetic	[84]
8	18.11	19.10	1,2-Benzenedicarboxylic acid, diisooctyl ester (**C**_**24**_**H**_**38**_**O**_**4**_)	Benzoic acid ester	Biopesticides, antibacterial	[106, 107]

MF: molecular formula; RT: retention time; PA: peak area.

## Data Availability

The data supporting the findings of this study are available from the corresponding author upon request.

## Abbreviations

AAJ: *Auricularia auricula judae*
CE: Chloroform extract
EE: 70% ethanol extract
FAs: Fatty acids
GC-MS: Gas chromatography mass spectrometry
HWE: Hot water extract
LA: Linolenic acid
LDL: Low-density lipoprotein
MF: Molecular formula
MX: *Microporus xanthopus*
PA: Peak area
PLA: Palmitic acid
RT: Retention time
TRE: *Trametes elegans*
TRV: *Trametes versicolor*
TU: *Termitomyces umkowaani*
